# Adapting response to a measles outbreak in a context of high vaccination and breakthrough cases: an example from Vaud, Switzerland, January to March 2024

**DOI:** 10.2807/1560-7917.ES.2024.29.22.2400275

**Published:** 2024-05-30

**Authors:** Alessandro Cassini, Ludovico Cobuccio, Emmanouil Glampedakis, Pascal Cherpillod, Pierre Alex Crisinel, Francisco-Javier Pérez-Rodríguez, Monica Attinger, Dominique Bachelin, Marie Nahimana Tessemo, Mirjam Maeusezahl, Céline Gardiol, Karim Boubaker

**Affiliations:** 1 Cantonal Doctor Office, Public Health Department, Canton of Vaud, Lausanne, Switzerland; 2 Infectious Diseases Service, Lausanne University Hospital, Lausanne, Switzerland; 3 Geneva Center for Emerging Viral Diseases, Geneva University Hospitals, Geneva, Switzerland; 4 National Measles and Rubella Reference Laboratory, Geneva University Hospitals, Geneva, Switzerland; 5 Laboratory of Virology, Laboratory Medicine Division, Geneva University Hospitals, Geneva, Switzerland; 6 Unit of paediatric infectious diseases and vaccinology, Service of Paediatrics, Women-Mother-Child Department, Lausanne University Hospital, Lausanne, Switzerland; 7 Communicable Disease Division, Federal Office of Public Health, Bern, Switzerland; * These authors contributed equally to this work and share first authorship.

**Keywords:** measles, breakthrough infections, vaccination, outbreak

## Abstract

A measles outbreak with 51 cases occurred in the canton of Vaud, Switzerland, between January and March 2024. The outbreak was triggered by an imported case, and 37 (72.5%) subsequent cases were previously vaccinated individuals. Epidemiological investigations showed that vaccinated measles cases were symptomatic and infectious. In a highly vaccinated population, it is important to raise awareness among healthcare professionals to suspect and test for measles virus when an outbreak is declared, irrespective of the vaccination status of the patients.

Switzerland pledged to eliminate measles as set by the World Health Organization (WHO) European Region [1]. In 2021, vaccination coverage with a measles-containing vaccine (MCV) in Switzerland was 98% for one dose and 96% for two doses in 16-year-olds [2]. Here we report on a measles outbreak in the canton of Vaud, Switzerland, between January and March 2024, triggered by an imported case and most subsequent cases were previously vaccinated individuals.

## Swiss measles surveillance and response

After a significant decrease by 98% between 2007 and 2018 [3], measles cases in recent years were either imported or linked to imported cases. The WHO concluded that endemic measles transmission was interrupted in Switzerland and an elimination status was reached in 2018 [3,4]. Since 2013, the Swiss Federal Office of Public Health (FOPH) has implemented national guidelines for responding to measles outbreaks, including case definitions [5], with the aim to ensure a consistent approach across cantons, which are ultimately responsible for the prevention and control of measles cases.

## Outbreak description

Between January and March 2024, the canton of Vaud responded to an outbreak of 50 measles cases linked to an imported unvaccinated case (index case), in a large university campus in the Lausanne region.

**Figure 1:**
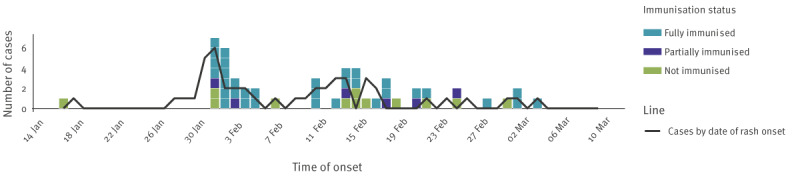
Timeline of a measles outbreak and immunisation status of cases, Canton of Vaud, Switzerland, January–March 2024 (n = 51)

After arriving in Switzerland, the index case consulted the university medical service on 15 January, was isolated and tested. On 16 January, the case developed a skin rash and measles virus (MeV) was confirmed by PCR. Twenty-one secondary cases were detected between 26 January and 3 February 2024 among people exposed at the same university (mostly students, a few visitors and personnel), and a further 16 were detected 5–26 February (Figure 1). The remaining 13 cases acquired the infection via additional transmission chains outside the university campus but were linked to cases in the university. Most cases were confirmed 1 day after development of a rash, although two cases did not develop a rash (Table). Considering exposures on the campus, the overall attack rate was ca 1% (37/3,700).

Fully immunised: received two doses of measles virus containing vaccine or reported previous measles; partially immunised: received one dose of measles virus containing vaccine.

**Table:** Methods used for confirmation of measles, presence of rash and vaccination status of cases in a measles outbreak, Canton of Vaud, Switzerland, January–March 2024 (n = 51)

Measles cases			
Presence of rash	Confirmatory method		
	PCR	Serology	Clinical symptoms and epidemiological link
Yes	45	3	1
No	2	0	0
Total	47	3	1
Vaccination status			
Unvaccinated	11		
1 dose	6		
2 doses	31		
Unknown	3		

The mean age of the cases was 24.3 years (range: 2–53 years), and 26 of them were male. Samples from 47 suspected cases were confirmed by PCR, three were confirmed by serology (IgM) (Table). Samples from one case were not tested, but the case had measles-related symptoms and an epidemiological link.

No complications or hospitalisations were recorded, and symptoms included fever, cough, conjunctivitis, coryza, headache, pharyngitis, myalgia and asthenia. Similarly to previous studies [6-9], milder symptoms were observed in those previously vaccinated (reported anecdotally from field investigations).

## Outbreak response

The university campus comprises around 4,000 students (average age of students: 23 years; proportion of female: 57%) from more than 120 countries. In January, ca 3,000 students and 700 staff were present on the campus. The epidemiological investigations and contact tracing activities by the Vaud cantonal public health authority revealed that the institution promotes an environment and pedagogical approach encouraging multiple interactions between students and staff. Hence, the entirety of the campus was considered as an exposure site and all students and staff were part of the contact tracing list.

Several emails were sent on 19 January to all students notifying them of the epidemic situation and requesting them to monitor their symptoms. On 2 February we offered vaccination catch-up and informed them about the closure of the campus until 19 February.

Due to secondary clusters, epidemiological investigations and control measures extended to household members, multinational companies hosting student internships and other universities.

## Virological investigations

All PCR-positive samples (n = 47) were sent to the national reference laboratory (Centre National de Référence pour la rougeole et la rubéole (CNRRR)) based at Geneva University Hospitals for confirmation and genotyping. The epidemiological link to the index case was laboratory-confirmed by the CNRRR for 44 of the 51 cases (three were impossible due to an insufficient viral load). For routine genotyping, WHO recommends sequencing a 450 nt region in the C-terminal N gene [10]. Sequences submitted to the WHO Global Measles Nt Sequence Database (MeaNS2, https://who-gmrln.org/means2) are assigned to a genotype and distinct sequence identifier (DSId) [11]. Viruses from all cases belonged to genotype B3 and were related to DSId 6418 (WHO named strain MVs/Quetta.PAK/44.20), except for four cases infected with DSId 6495 (mutations occurring at least twice) and one with DSId 8778. The 6495 and 8778 variants differed by 1 nt from 6418 and most probably mutated from the latter, given the short genetic distance and the confirmed epidemiological links. To our knowledge, this is the first time that the variant 8778 has been identified. The sequences of the three DSIds have been deposited in GenBank [12] (accession numbers: PP534414-PP534416). Our index case represents the first time this variant has been detected in Switzerland.

## Transmission chains, vaccination status and adapting the response protocol

**Figure 2:**
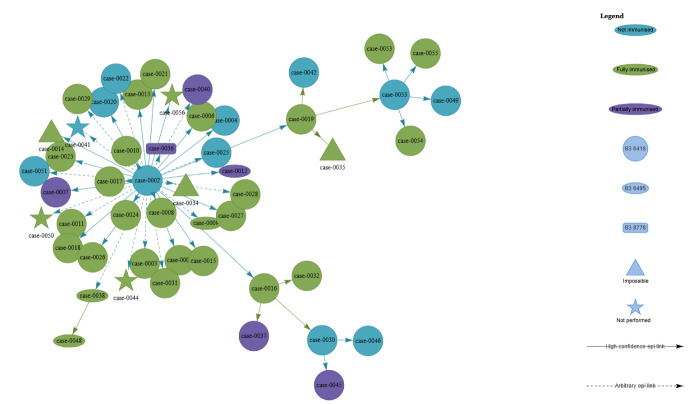
Transmission chains between cases in a measles outbreak, Canton of Vaud, Switzerland, January–March 2024 (n = 51)^a^

Transmission chains were illustrated based on epidemiological investigations and laboratory confirmation (Figure 2; with interactive page). Immunity to MeV was assessed based on provision of a verified vaccination card or proven history of disease. Most cases (n = 31) had received two doses of MCV, six had received one dose, 11 were unvaccinated, and three had an unknown vaccination status (Table). The number of breakthrough cases was 37 (72.5%), considering at least one MCV or previous infection.

The interactive chains of transmission can be seen at: https://leskargot.github.io/outbreak/2024/03/31/rougeole.html.

On at least two occasions, it appeared that double-vaccinated individuals infected other persons (Case 0016 and 0019 in the transmission chain). Five of 12 subsequent cases were vaccinated with two MCV doses and two had one MCV dose.

**Figure 3:**
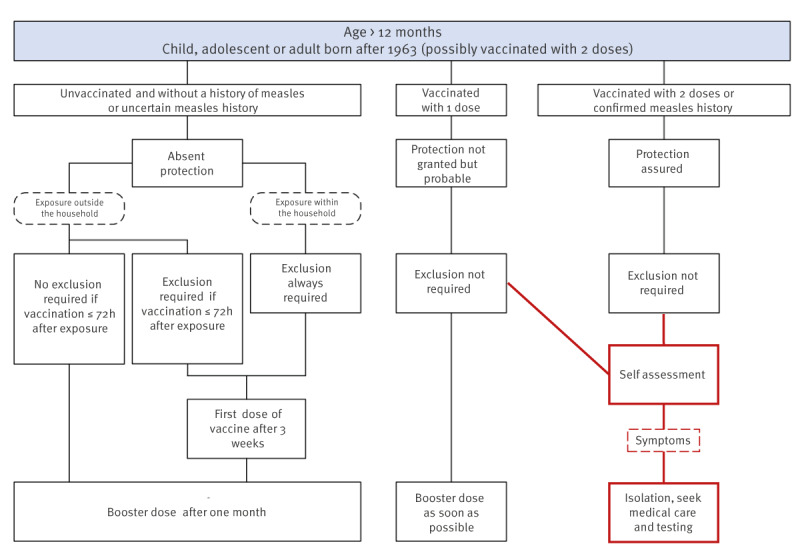
Flowchart of management of a person (aged > 12 months) exposed to a case of measles in a measles outbreak, Canton of Vaud, Switzerland, January–March 2024

The Swiss protocol for the control and management of measles cases and outbreaks [5] was reviewed and adapted in the light of the evidence discussed above. Regardless of their immunity towards MeV, contacts were asked to closely monitor their symptoms. If any appeared, they were instructed to isolate, contact the response team (cantonal doctor’s office) and seek testing (Figure 3, in red the adaptation of the algorithm). In parallel, several communications were sent to physicians at all care levels, pharmacists and other healthcare workers to raise awareness on the need to test for MeV when symptoms appeared, regardless of vaccination status, and apply infection prevention and control airborne precautions when suspecting measles.

Adaptation from the general procedure marked in red.

**Figure 4:**
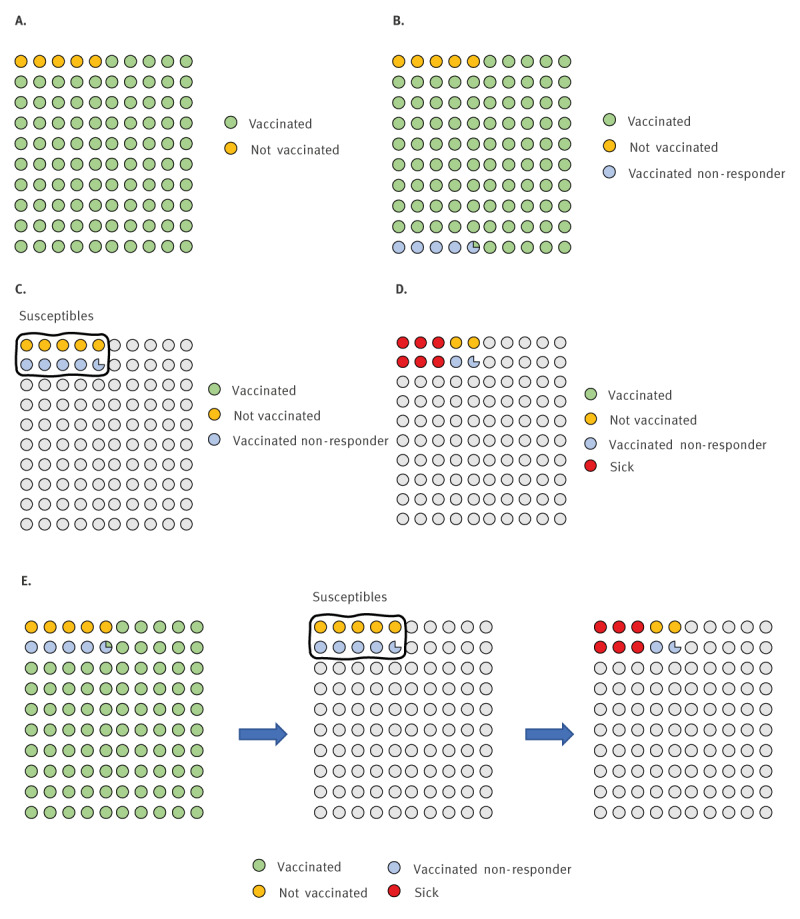
Illustration of cases in a highly vaccinated population

In 1985, Orenstein et al. postulated that in a highly vaccinated population with a highly effective vaccine, it is relatively common to expect an important proportion of cases among those fully vaccinated [13]. An animation of Orenstein’s paradox is presented in Figure 4 and in Supplementary Material.

## Discussion

Epidemiological and molecular information indicated that, of the 51 measles cases, 50 were secondary to an imported unvaccinated case. The outbreak of measles experienced in the canton of Vaud was striking for the high proportion of breakthrough cases (37/51; > 70%) and because previously vaccinated individuals further infected vaccinated and unvaccinated persons.

Published studies reporting measles outbreaks with differing proportions of breakthrough cases vary widely. In Japan, this ranged from 88%, based on the presence of measles-specific IgG [14], to 75% for at least one dose or 18.8% for two doses [15]. In Sweden, 50% and 57% of cases were vaccinated with two doses and one dose, respectively [9], in Spain up to 14% of cases were vaccinated with two doses [7], in the US 9% and 11% of individuals were vaccinated with one and two doses, respectively [6], and in Northern Italy 7% of cases were breakthrough [16].

Although the risk of transmission of MeV from vaccinated cases is deemed low [6,9,17,18], the scientific literature has been increasingly reporting transmission from vaccinees [15,16,19-21], similar to our outbreak report (two individuals vaccinated with two doses infecting six people). Particularly in settings of sustained measles elimination, increasing evidence underlines the need to closely monitor the symptoms of exposed individuals, as well as testing and isolation if symptoms develop, irrespective of vaccination status [15,19,20].

Exposure in a closed environment may play a role in transmission, regardless of vaccination status. For example, case 0016 visited a small medical centre while symptomatic, exposing two patients in the waiting room, twelve additional patients who attended the same rooms, and four healthcare workers. Despite a universal face mask policy in place in the facility at the time of the consultation, case 0016 infected three cases, resulting in an attack rate of 16.7%, much higher than the 1% observed in the university campus. However, this remains comparatively lower than similar high-level exposure settings, such as households where attack rates can reach 90% in non-immune persons [22].

The age at which infants should receive their first vaccine has been debated since the availability of MCV, including the WHO Strategic Advisory Group of Experts (SAGE) on immunisation [23]. In a recent measles outbreak in a French secondary school involving 64 teenagers, 60% were double-vaccinated, and of those, 73% had received their first dose before 12 months of age [24]. The attack rate among adolescents who received MCV1 before 12 months of age was higher (10% vs 3%). This led the French response team to recommend a third booster dose. However, our cohort had received MCV1 after the age of 12 months. Moreover, a recent systematic review did not find any significant differences between infants receiving MCV1 before or after 9 months of age [25]. Therefore, we deemed a booster MCV3 campaign unnecessary.

Although immunity evasion was not possible to measure (the virus did not grow on culture and it was not possible to take blood samples), we did not consider this as a driver for the high proportion of vaccinated measles cases and their ability to infect.

When applying the formula developed by Orenstein et al. [13] to the situation of the population affected by the measles outbreak, which has 96% MCV coverage and assuming 95% effectiveness of MCVs, one can predict that 55% of cases will be fully vaccinated, similar to what was observed during this outbreak (60.8%).

## Conclusion

The outbreak ended in early March, 7 weeks after the first case was diagnosed. Herd immunity and the measures put in place to respond to the outbreak and described in this Rapid communication (early detection, isolation, contact tracing, monitoring of symptoms, tailored information and communication) were effective. The impact of the measles outbreak, both in terms of severity of the disease and number of cases, was low given the number of exposed people and opportunities for disease transmission: the outcome highlights the effectiveness and importance of vaccination against measles. Conversely, the high effectiveness of MCV is confirmed by the formula for the indirect estimation of vaccine effectiveness. In a context of measles elimination, a significant proportion of breakthrough infections with the ability to further transmit should be expected. Response guidelines should include monitoring and testing of symptomatic individuals, regardless of vaccinations status. After declaring an outbreak, awareness on testing all symptomatic individuals should be raised among doctors and healthcare centres.

## Supplementary Data

94000 Supplementary Material
