# Supplementary material for: Adapting response to a measles outbreak in a context of high vaccination and breakthrough cases: an example from Vaud, Switzerland, January to March 2024
Source: Euro Surveill. 2024 May 30;29(22):2400275. doi: 10.2807/1560-7917.ES.2024.29.22.2400275 (PMC11141130; doi:10.2807/1560-7917.ES.2024.29.22.2400275)
Supplement: Supplementary Material [file 24-00275_CASSINI_Supplement.pptx]

## Slide 1
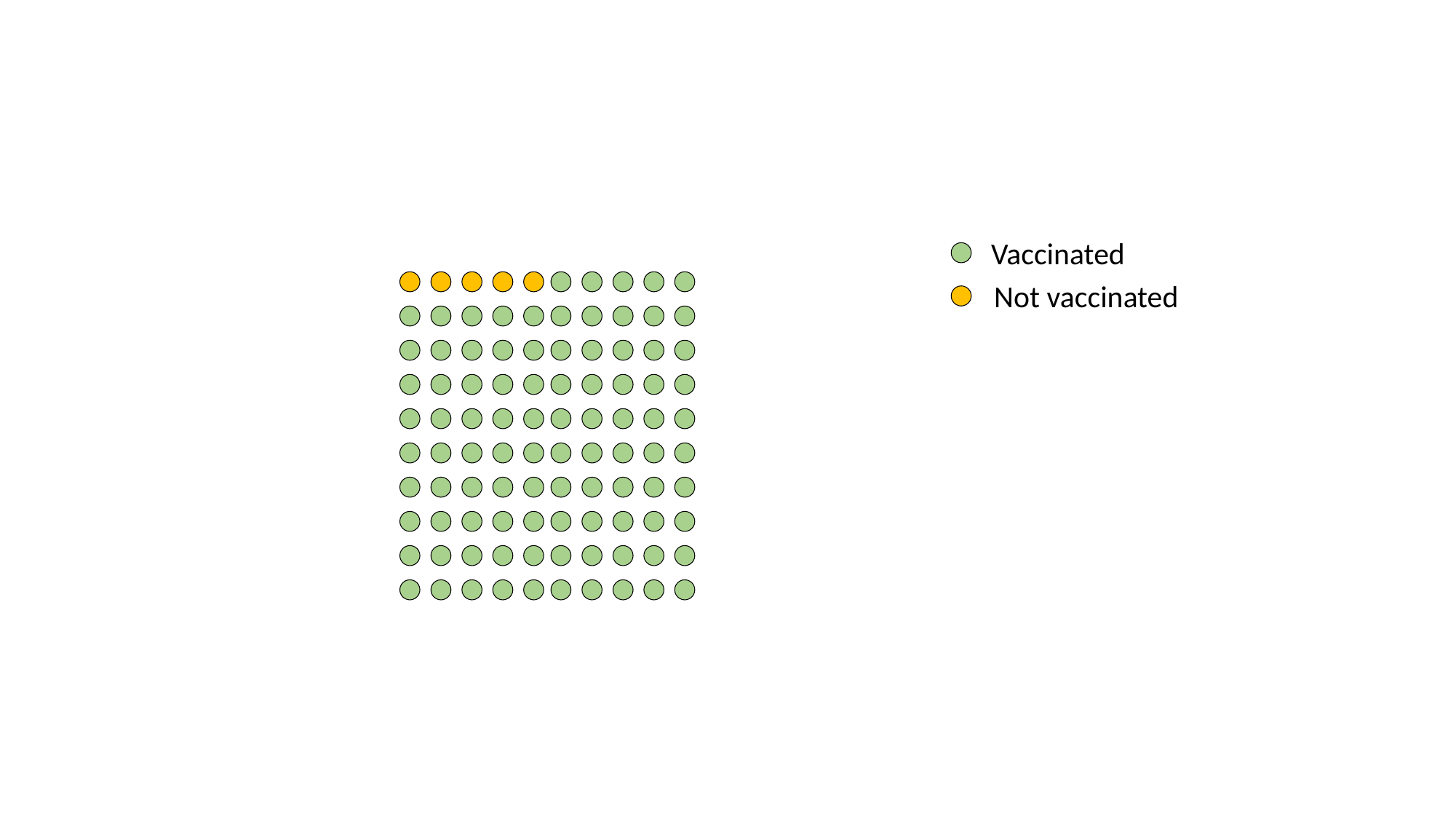

Vaccinated
Not vaccinated

## Slide 2
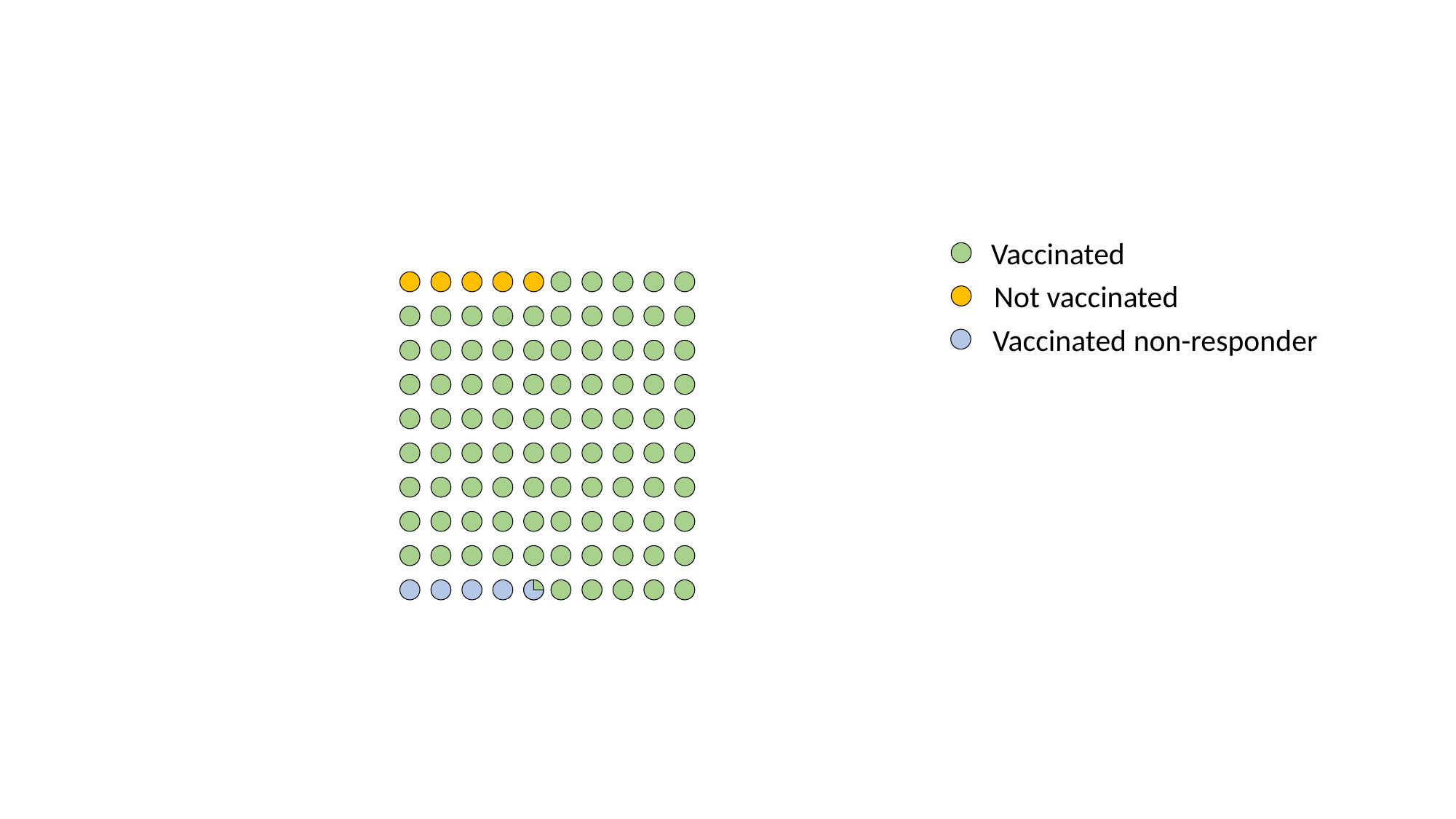

Vaccinated
Not vaccinated
Vaccinated non-responder

## Slide 3
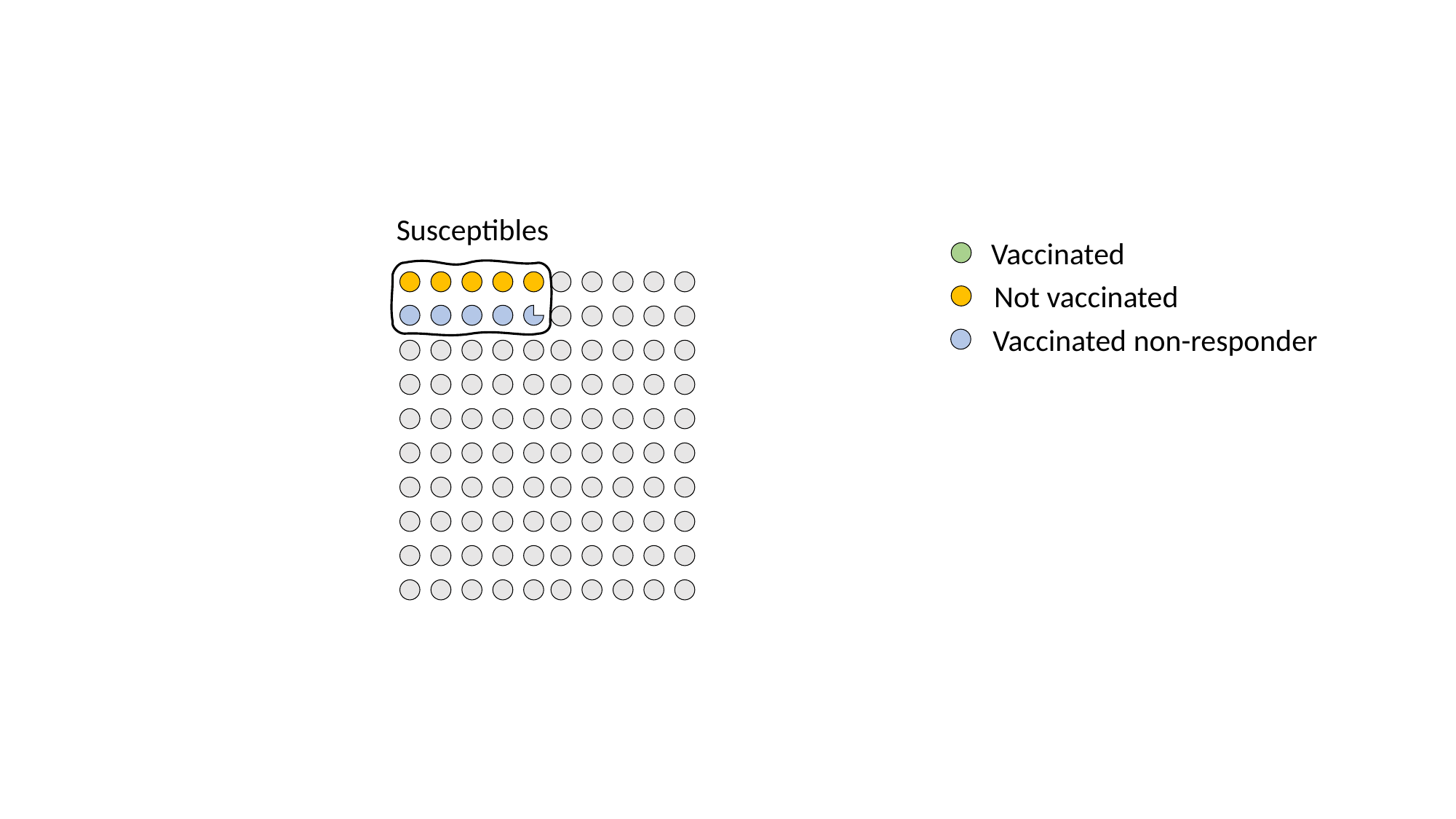

Susceptibles
Vaccinated
Not vaccinated
Vaccinated non-responder

## Slide 4
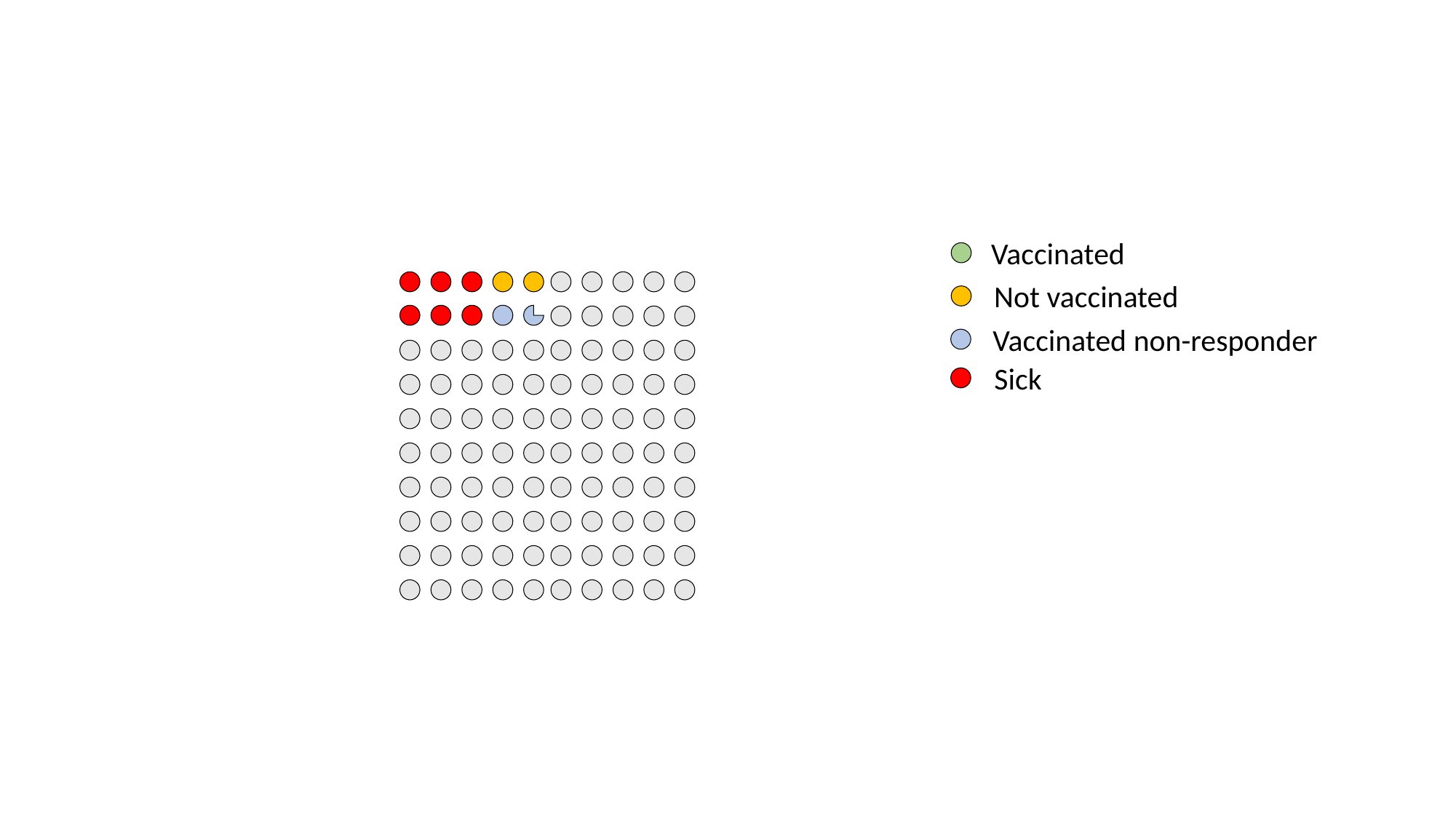

Vaccinated
Not vaccinated
Vaccinated non-responder
Sick

## Slide 5
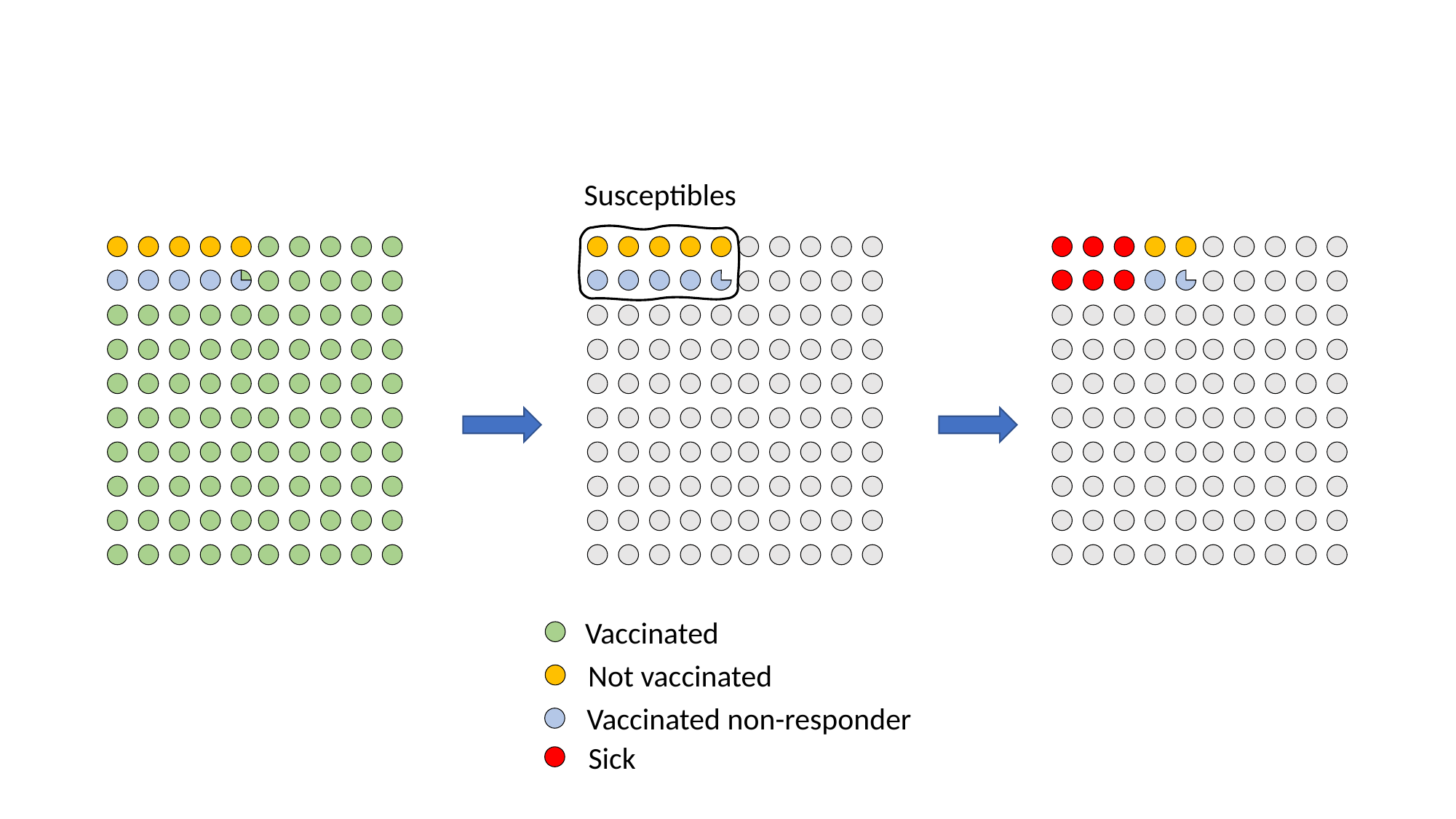

Susceptibles
Vaccinated
Not vaccinated
Vaccinated non-responder
Sick
